# A Systematic Review of Conceptualizations, Early Indicators, and Educational Provisions for Intellectual Precocity

**DOI:** 10.3390/jintelligence12080076

**Published:** 2024-08-02

**Authors:** María Leonor Conejeros-Solar, Sandra Catalán, María Paz Gómez-Arizaga, Tatiana López-Jiménez, Natalie Contador, Katia Sandoval-Rodríguez, Cristóbal Bustamante, Josefa Quijanes

**Affiliations:** 1 Escuela de Pedagogía, Pontificia Universidad Católica de Valparaíso, Valparaíso 2362807, Chile; sandra.catalan@pucv.cl (S.C.); tatiana.lopez@pucv.cl (T.L.-J.); natalie.contador@gmail.com (N.C.); katia.sandoval@pucv.cl (K.S.-R.); cristobal.bustamante.silva@gmail.com (C.B.); josefa.quijanes@usach.cl (J.Q.); 2 Escuela de Psicología, Universidad de Santiago de Chile, Santiago 9170022, Chile; maria.gomez.ar@usach.cl

**Keywords:** intellectual precocity, giftedness, early indicators, educational programs, systematic review

## Abstract

Intellectual precocity in children poses unique challenges and opportunities for educational systems. This systematic review aims to comprehensively analyze intellectual precocity in children until 6 years old, including its definition, manifestations, and various educational programs for intellectually precocious learners. Following PRISMA guidelines, a comprehensive search of electronic databases was conducted. The study included 26 articles published between 2013 and 2023 that provided a conceptualization of precocity or giftedness, and/or focused on characteristics of precocity, and/or investigated educational programs for intellectually precocious children. The authors’ conceptualizations of precocity varied, with some providing clear definitions based on a developmental view of precocity, while others merely mentioned the concept. Early indicators of superior traits have been observed in areas such as reading, math, problem-solving, and even in fields that have been traditionally disregarded, such as visual arts. Educational provisions varied widely, including approaches based on enrichment and project-based learning; however, interventions based on socioemotional elements are also highlighted. The findings emphasize the importance of early identification and targeted educational strategies to support the unique needs of intellectually precocious individuals. Future research should focus on longitudinal studies and the development of evidence-based interventions.

## 1. Introduction

Giftedness as an overall construct is a complex and dynamic phenomenon that is culturally situated and contextualized (Carrillo 2013; Ngara 2008; Plucker et al. 2021). There is no broad consensus on its definition, as evidenced by the diversity of models that have emerged over time (Matthews and Jolly 2022; Sternberg and Ophelie 2022). Current approaches conceptualize giftedness as a developmental process (Gagné 2015, 2021; Pergantis 2024; Renzulli 2011; Subotnik et al. 2019). This view suggests that abilities are malleable, so the development of giftedness should be considered a process that unfolds over time, with critical transition points requiring comprehensive and timely responses for gifted individuals (Subotnik et al. 2011).

For this review, the term “intellectual precocity” (IP) will be used, a concept situated within the framework of giftedness that refers to the early and accelerated development of linguistic, psychomotor, cognitive, and socioemotional skills in children of the same age range and under equal social, ethnic, and cultural conditions (Al-Hroub and El Khoury 2018; Albes et al. 2013; Jabůrek et al. 2021). At the cognitive level, Lubinski and Benbow (2021) have reported the presence of general intellectual ability and three specific traits that are essential for this group: mathematical, spatial, and verbal abilities. Other authors, such as Villanueva García (2011), support these findings, indicating that the most commonly observed early skills are related to mathematical and verbal patterns, identifying key characteristics such as (a) the ability to quickly understand mathematical operations; (b) the ability to express oneself using a rich and varied vocabulary; and (c) the inclination to seek explanations and reasoned answers. Gómez-León (2020) highlights creativity, abstract thinking, and critical thinking as central traits in IP learners. IP has also been considered in the study of executive functions, where a greater development of these skills has been observed compared to children of the same age (Sastre-Riba and Castelló-Tarrida 2017). Lastly, asynchrony, a concept coined by the Columbus Group in 1991 to qualitatively describe the experiences of precocious youth, refers to an uneven development between cognitive, emotional, social, and physical areas. This discrepancy can lead to increased complexity, intensity, heightened awareness, social alienation, and vulnerability, which must be considered in understanding IP (Silverman 1997).

For this review, we assume Gagné’s (2021) Differentiating Model of Talent Development (DMGT) as a conceptual framework, which, although it does not specifically refer to IP, considers the intellectual realm as one of the domains in which high ability might present itself. According to this model, natural ability (called aptitudes) in the intellectual dimension places gifted children in the top 10% of their same-age peer group. Through developmental and learning processes, both formal and informal, these abilities can be transformed into systematically developed talents or skills. This process is facilitated by two types of catalysts: intrapersonal (such as motivation and temperament) and environmental (e.g., family and school), which actively moderate talent development. The dynamic interaction of these components can foster or sometimes hinder the emergence of talent (called competencies) in at least one ability domain. Gagné asserts that natural abilities are not innate but develop throughout life, especially in a person’s early years. Consequently, the environmental aspects and early learning experiences to which IP learners are exposed are critical to their development.

Unfortunately, IP is one of the most neglected areas in the broad field of giftedness (Hertzog et al. 2018; Jolly and Kettler 2008; Martins and Chacon 2016; Walsh 2014). This is problematic considering that the first years of life are crucial for later cognitive, emotional, and social development (Kaplan and Hertzog 2016), especially for young gifted learners who begin their educational journey with already acquired learning but do not receive a differentiated curriculum (Conejeros-Solar and Gómez-Arizaga 2020; Pfeiffer 2017). Studies suggest that professionals at this educational level assume that these children will be served later in their school education (Wellisch 2019). Additionally, they may not be adequately prepared to identify these children and/or design learning opportunities tailored to their needs, resulting in practices that are not sufficiently adapted to the needs of these children (Grant and Morrissey 2019). As a result, pedagogical practices may be routine, inflexible, and unchallenging (Kettler et al. 2017a, 2017b). 

Sternberg (2024) highlights the importance of considering that the development of an individual as ‘gifted’ is always a collective process that occurs within local sociocultural and temporal contexts. When the educational needs of precocious children are not met, they may become disenchanted with learning, experience boredom, seek inappropriate attention from their educators, and exhibit lower psychological well-being, which clearly constitutes a problem for their development (Kroesbergen et al. 2016; Mathijssen et al. 2021). Research indicates that teaching, family, and social support are crucial for enhancing the skills of IP learners and developing educational processes tailored to their needs, interests, and motivations (Belur and Oğuz-Duran 2017; García-Cepero and Iglesias Velasco 2020). This support is even more relevant in certain contexts since early and quality educational responses become more important for gifted young children from economically disadvantaged and culturally diverse backgrounds (Grant and Morrissey 2019). Failure to develop early skills in a timely manner risks leading to underachievement and dropout, which can have negative consequences for both children and society (Hoogeveen 2022).

Educational practices can significantly impact IP learners (Pfeiffer 2017). Internationally, acceleration practices are suggested, allowing children to continue participating in learning experiences in the school context with an accelerated promotion of the educational level appropriate to their intellectual and social needs (Wellisch 2019). Curricular strategies are proposed to make content-knowledge more complex and enriched, favoring methodologies that encourage intellectual curiosity and the development of social and emotional imagination (Gotlieb et al. 2016). They also include challenging activities that promote reflection and the development of critical and creative thinking (Gómez-León 2020).

Identifying and recognizing IP learners at the educational level is the first step toward providing comprehensive care for them (Karabulut and Ömeroğlu 2021). However, the lack of focus on precocious learners has resulted in IP being a scarcely studied area, with few policies and financial support. Additionally, there is a prevalence of myths, such as the belief that these children will succeed on their own without the need for appropriate services (Chamberlin et al. 2007). In this context, this systematic review aims to synthesize the existing literature on three core aspects: (a) conceptualizations of precocity that underpin the research, (b) early indicators of IP, and (c) types and modalities of educational programs for IP learners. 

## 2. Method

To comprehensively address the research objectives, the PRISMA (Preferred Reporting Items for Systematic Reviews and Meta-Analyses) guidelines were used, providing a systematic and structured approach to conducting literature reviews. This model has been utilized to improve the transparency and quality of such studies by establishing clear guidelines for the identification, selection, evaluation, and synthesis of the available scientific evidence (Page et al. 2021; Shamseer et al. 2015). Following these guidelines, a protocol was developed to plan the review and analysis process, which followed five stages: (1) definition of research objectives; (2) search process; (3) definition of inclusion and exclusion criteria; (4) data selection and extraction process; and (5) data analysis.

**Phase 1: Research objectives**

To analyze the documents on IP published in the last 10 years, three research objectives were set: (1) to examine the conceptual foundations underlying current research on IP; (2) to identify the distinctive characteristics of learners with IP; and (3) to explore the programs used to serve young gifted children. These objectives provide an overview of the situation regarding the understanding and approaches to IP in an underexplored field.

**Phase 2: Search Process**

The review focused on peer reviewed scientific studies published in high-impact journals and available online, searching for articles in two databases: SCOPUS and Web of Science (WoS). These databases were selected for their rigor and comprehensiveness, integrating all relevant sources for basic and applied research (Huanca-Arohuanca 2022).

To identify potentially eligible studies, sets of keywords were defined to carry out the article search process rigorously. The following Boolean terms were used: (“gifted young children” [All Fields]) OR (“young gifted learner” [All Fields]) OR (“young gifted children” [All Fields] OR (“intellectual precocity” [All Fields]) AND (“characteristics” [All Fields]) AND (“programs” [All Fields]). The search was limited to articles published in English or Spanish, focusing on these two languages due to the scope and visibility of publications. Nearly 95% of the literature is in English (Torres 2017), and Spanish is the native language of the authors of this review.

The search considered articles published from 2013 to 2023, a 10-year period that captures the most recent advances and emerging trends in the field, ensuring that the review is relevant and up-to-date (Guirao 2015).

**Phase 3: Definition of Inclusion and Exclusion Criteria**

After conducting the search, 149 studies were identified. Relevant information for each article, including authors, title, keywords, abstract, Digital Object Identifiers (DOIs), year, and journal of publication, was recorded in an Excel database. Two researchers manually reviewed this database to identify duplicates, resulting in the exclusion of 25 articles due to duplication, leaving 124 articles for further analysis.

Inclusion and exclusion criteria were established to ensure that the selected articles aligned with the review’s objectives and scope. Inclusion criteria ensured relevance to the review’s focus, while exclusion criteria discarded studies that did not meet strict methodological standards or addressed topics outside the field of interest.

The study’s inclusion and exclusion criteria were divided into three categories: (a) participants, referring to the specific characteristics of the sample (children, teachers, and parents) considered eligible for the study; (b) concept, which considered definitions of IP, its characteristics, and the conceptual frameworks used; and (c) context, which referred to the educational settings where the research was conducted. Please refer to Table 1 for further details.

A comprehensive evaluation of the articles was conducted by reading their titles, keywords, and abstracts. Five researchers rigorously and independently applied the predefined set of inclusion and exclusion criteria, scoring each article. Articles requiring collective decisions were reviewed in group meetings.

To be included in the review, the study needed to meet at least one criterion in each category.

**Phase 4: Data Selection and Extraction Process**

In the first review of titles, abstracts, and keywords incorporating the inclusion and exclusion criteria, 96 studies were excluded from the initial 124. Of these, 58 were excluded because they did not refer to gifted children aged 6 years or younger, and 6 did not refer to educators teaching these children at the preschool level.

Regarding the concept category, 10 articles were excluded because they did not refer to the conceptualization, characteristics, or educational programs of IP children, focusing instead on existing policies and legislation.

Regarding the context category, 22 studies were discarded because they referred to research not conducted in formal or informal educational contexts, focusing instead on socioemotional interventions.

**Figure 1:**
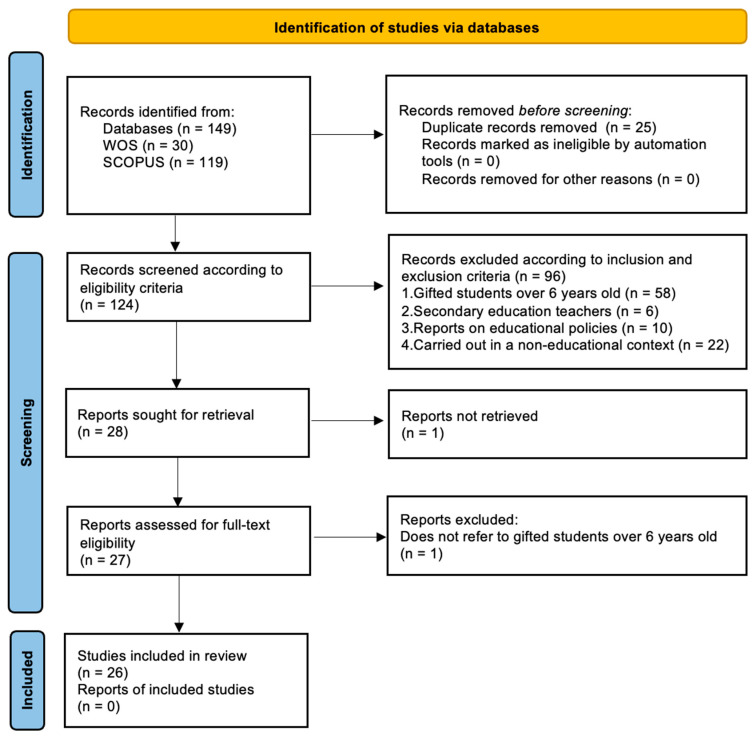
PRISMA flow diagram describing the results of the literature review adapted from Page et al. (2021).

Of the remaining 28 articles, a full-text reading was performed. One article was discarded because the full text was unavailable, and another was excluded because it did not explicitly address gifted children under 6 years old. This left 26 articles for the systematic review, as presented in Figure 1.

**Phase 5: Data analysis**

To extract data from the publications, a qualitative content analysis approach was used, emphasizing understanding and providing knowledge of the phenomenon under study (Roller and Lavrakas 2015). The content was analyzed through a systematic process of classification to identify categories and themes describing the research phenomenon (Assarroudi et al. 2018; Roller and Lavrakas 2015). Predefined categories were used based on the research objectives: conceptualizations of giftedness and precocity, characteristics of IP, and programs and modalities of educational interventions.

Six researchers participated, organized in pairs to ensure the reliability of the analysis. Initially, tables were created for each publication, collecting sections of the texts that responded to the formulated categories. Subsequently, the data were organized, reconstructed, and managed into codes and subcategories, allowing for a deeper analysis (Roller and Lavrakas 2015). Before writing the results, the subcategories were analyzed by the entire research team, discussing their relevance, coherence, and the most appropriate names. Table 2 shows this organization.

## 3. Integrative Review of the Literature

Data extracted from the studies, including the title, author/year of publication, journal location, method, and participants, were compiled into a table after selecting eligible studies. The studies used can be found in Table 3.

### 3.1. Conceptualizations of Giftedness and Precocity

There is a general agreement in the current literature that giftedness should be understood and conceptualized as a complex and multifaceted construct (Zenasni et al. 2016). This perspective implies a shift from a conservative to a more inclusive approach that considers the environment as a crucial element (Renzulli 2002; Smedsrud 2020).

Empirical studies, however, do not always defend or adhere to a single definition of giftedness, often providing only a broad discussion of the construct, which can be a historical overview or a synthesis of the main models in the field and their components. Many studies refer generically to the concept without committing to a particular definition. Regarding the first research objective related to the conceptualizations of precocity in the reviewed articles, out of the 26 studies included in this review, 21 provided definitions of giftedness. It is important to mention that the articles do not refer to precocity in particular as a unique phenomenon within the framework of giftedness but tend to provide broad definitions that do not necessarily point to this phenomenon, which is exclusive to early childhood. Therefore, it was not possible to refer only to the concept of IP in the articles reviewed, and it was necessary for this research objective to expand to the concept of giftedness.

**Giftedness as Abilities or Traits**

Eleven articles presented a conceptualization of giftedness that consists of two components: (a) possessing characteristics superior to the reference group and (b) expressing these attributes in one or more domains. Even if the domains can vary widely (e.g., art, language, leadership, creativity), some authors limit giftedness only to the cognitive/intellectual realm (e.g., Jabůrek et al. 2021). Few authors provided explanations for characteristics that form the basis of higher abilities. For instance, Papadopoulos (2021) linked giftedness with neuropsychological characteristics, such as a more rapid and efficient neural system.

Interestingly, only two articles referred to the combination of cognitive traits with psychological/intrapersonal characteristics, providing a more qualitative and subjective perspective on the internal experience of being gifted (i.e., Daǧlioǧlu and Suveren 2013; Renati et al. 2023). The absence of socioemotional skills in the trait definitions is striking. This is especially notable considering that in the study of IP, the relationship between early academic skills and socioemotional traits, such as self-regulation, has been examined in depth (Gözüm and Aktulun 2021).

Only one study (Firat and Bildiren 2023) referred to the traits that can be found in twice-exceptional (2e) students, providing a combination of characteristics that emerge both from giftedness and disabilities. This low number of investigations is understandable because not all studies focused on this manifestation of giftedness but rather on the general population of gifted young children. However, it is relevant to mention that twice-exceptionality is an understudied field in early childhood and IP (Chamberlin et al. 2007).

Fewer authors referred to precocity to provide a more specific definition of the concept. The studies that do so briefly state the main characteristics or traits observed in IP learners, such as advanced abilities in language, memory, and mathematics (e.g., Bildiren and Kargın 2019; Mollenkopf et al. 2021).

**Broad Definition of Giftedness**

As previously mentioned, in some articles (n = 7), authors attempted to refer to the concept of giftedness but only at a discussion level, addressing the complexity of the construct without necessarily committing to a single definition. For example, some studies referred to existing models of giftedness (e.g., Artiles 2022), while others discussed the definition and/or evolution of the concept (e.g., Mohamed and Elhoweris 2022). Some authors (e.g., Villuendas-Rey et al. 2020) addressed the complexities underlying the definition of giftedness, particularly in relation to the identification process, without advocating for a specific view or model.

Providing a discussion on the topic of giftedness is necessary due to the lack of consensus on the topic. This can be seen in the varying definitions and methods used to measure giftedness, such as through intelligence or performance instruments (Hernández-Torrano and Kuzhabekova 2020). However, it is critical to adhere to particular definitions and/or models of giftedness with the purpose of (a) providing a better articulation of the research objectives that must focus on IP and (b) having a solid conceptual basis when addressing other relevant elements in IP, such as identification processes, interventions, and curriculum, among others. A specific approach can provide room for a multidimensional analysis of all aspects of the phenomenon and its long-term impact.

**Giftedness as a Multifactorial/developmental Construct**

Finally, only three studies addressed the concept of giftedness from a holistic perspective, emphasizing the interplay between traits and context to understand the manifestations of giftedness. These studies not only emphasize the understanding of giftedness from a psychosocial perspective (Papadopoulos 2020b) but also the importance of incorporating several sources for its measurement and the critical role of the environment in promoting the development of potential (Podobnik and Selan 2021). Conceptualizing IP as a developmental construct with a strong environmental component is critical to understanding a phenomenon that is more than the sum of its characteristics. It is in the interaction with the environment that gifted behaviors appear (Renzulli 2011), and it is in the educational environment where skills and traits need to emerge in order to nurture them adequately.

### 3.2. Characteristics of Intellectual Precocity

In relation to the question regarding the characteristics of IP, it is important to note that there are no two children with the same qualities or traits, as there is as much diversity in this group as in all other children (Artiles 2022). The total number of selected articles (n = 26) generally described the characteristics of IP, noting that this condition implies advanced cognitive abilities and asynchronous development.

The analyzed articles are emphatic in pointing out that IP learners may show, among other things, advanced linguistic and reasoning skills compared to their peers of the same age, rapid understanding and learning, and insatiable and intense curiosity (Jabůrek et al. 2021; Firat and Bildiren 2023; Villuendas-Rey et al. 2020). In addition, five developmental domains related to IP are presented as follows in the reviewed studies:

**Cognitive Characteristics**

Regarding the specific characteristics of cognitive development, the most recurrent ideas in the literature analyzed will be presented. Intellectual precocity implies advanced cognitive abilities and higher intensity (Daǧlioǧlu and Suveren 2013). On the other hand, Jawabreh et al. (2022) highlight in IP learners a capacity for curiosity that is different from what is expected for their age, as well as outstanding abilities in executive functioning, especially in working memory, cognitive flexibility, inhibitory control, and planning (Sofologi et al. 2022).

It should be noted that while three articles make more specific characteristics of cognitive development explicit (Firat and Bildiren 2023; Sofologi et al. 2022; Cosar et al. 2015), most point out that young gifted children show sustained attention and deep perception, implying sharper and more detailed observation. They are also noted for faster learning, the ability to formulate questions, problem-solving, and sustained focus at an early age.

**Motor Skills**

Regarding the motor skills associated with IP, the analysis found that most of the articles (n = 23) explicitly state one or more characteristics in this area of development. Some general characteristics are described in which advanced fine and gross motor skills tend to stand out (Daǧlioǧlu and Suveren 2013; Jawabreh et al. 2022; Firat and Bildiren 2023; Sofologi et al. 2022; Hernández-Torrano and Kuzhabekova 2020; Villuendas-Rey et al. 2020; Kamphorst et al. 2021). Similarly, fine motor skills are very refined (Podobnik and Selan 2021), showing excellent agility (Darga and Ataman 2021) and more advanced motor (Jabůrek et al. 2021) and visual–motor coordination than expected for their age (Papadopoulos (2020a, 2020b).

Specifically, in terms of gross motor development, there is a significant difference in crawling, walking, and scooting (Mollenkopf et al. 2021; Bildiren and Kargın 2019). Some of the articles (n = 7) (Papadopoulos 2020a, 2020b; Dereli and Deli 2022; Renati et al. 2023; Antoun et al. 2022; Aldosari 2023) point out that IP is not directly related to specific motor characteristics; however, one article reports the importance of advanced motor development in the achievement of academic skills (Kamphorst et al. 2021).

**Linguistic Development**

Another relevant ability reported in some articles (n = 12) refers to language skills and the significant development of communication skills in IP learners (Firat and Bildiren 2023; Mohamed and Elhoweris 2022; Artiles 2022; Mollenkopf et al. 2021; Gözüm and Aktulun 2021).

Regarding the above, this group of children often show early language development (Mohamed and Elhoweris 2022; Papadopoulos 2020a; Bildiren and Kargın 2019). They use long and complex sentences, process large amounts of vocabulary, understand verbal analogies, express themselves fluently, and access the reading process earlier than their age-matched peers (Renati et al. 2023; Jawabreh et al. 2022; Sofologi et al. 2022; Jabůrek et al. 2021).

According to Dereli and Deli (2022), precocious learners use abstract concepts in sentences, and sentences used by them are longer and more complex; they acquire the mother tongue more quickly and can engage in extensive discussions on various topics.

Additionally, Gözüm and Aktulun (2021) report that linguistic experiences provide children with support for the development of self-regulation skills. With the development of self-regulation skills, language skills are addressed under control, and children ask questions, creating problems and situations in their minds to find answers to their own problems.

**Artistic/creativity Skills**

Regarding artistic and creative skills, most studies point out that both skills are manifested in IP learners. Specifically, some studies (n = 9) highlight artistic expression, such as music enjoyment and sensitivity to rhythms, as well as faster learning when playing instruments (Daǧlioǧlu and Suveren 2013; Firat and Bildiren 2023). In addition, they highlight advanced artistic ability compared to other children their age (Dereli and Deli 2022; Mohamed and Elhoweris 2022; Aldosari 2023). They demonstrate novel approaches and expression of visual and spatial skills at a higher level of development (Darga and Ataman 2021). Artistic skills are performed exceptionally well (Artiles 2022), including a vivid imagination and extensive use of abstract thinking (Renati et al. 2023).

On the other hand, creativity stands out as a highly valued skill in the examined literature (n = 12). It is often associated with children who exhibit unique thinking patterns, express themselves freely, possess vivid imaginations, and demonstrate originality and high sensitivity (Podobnik and Selan 2021). Additionally, they tend to generate new solutions and ideas at an early age (Jawabreh et al. 2022; Bildiren and Kargın 2019; Papadopoulos 2020a; Jabůrek et al. 2021). As well as an outstanding ability in art (Mohamed and Elhoweris 2022), especially in painting and music, they also use more non-verbal language, including gestures and mimicry (Firat and Bildiren 2023; Cosar et al. 2015). Finally, six studies (e.g., Kamphorst et al. 2021; Sofologi et al. 2022) did not assess these types of skills in IP learners.

**Socioemotional Skills**

Based on the performed review (n = 26), it was found that children with IP exhibit distinct social–emotional development when compared to their peers of the same age. Some authors (n = 12) have reported that infants are characterized by greater emotional sensitivity, intensity, complexity, and intense curiosity (Sofologi et al. 2022; Mohamed and Elhoweris 2022; Podobnik and Selan 2021). Similarly, four of the articles refer to the asynchronous development observed between cognitive skills and socioemotional development (Dereli and Deli 2022; Sofologi et al. 2022; Firat and Bildiren 2023).

In one article, preschool teachers noted that this group of children may exhibit advanced socioemotional skills and achieve satisfactory social relationships with peers, sharing friends and an interest in social issues (Jawabreh et al. 2022). However, given these advanced skills, they may experience difficulties in interacting with others by expressing an exaggerated sense of self-esteem or hypersensitivity, which may hinder their ability to connect with others (Mohamed and Elhoweris 2022). Furthermore, one study (Firat and Bildiren 2023) reported that they are characterized by their empathy, motivation, high self-confidence, and leadership in leading groups from an early age.

In summary, based on the analysis of the corpus of articles related to the characteristics of children with IP, these children exhibit behaviors and skills that develop early and systematically at a higher level than their peers of the same age. These skills can be found across various abilities, disciplines, and areas of development, with a higher prevalence of intellectual and linguistic characteristics.

### 3.3. Types and Modalities of Educational Programs

Appropriate services for the IP preschool child must take into account both the child’s asynchronous development and his/her emerging skills (Cukierkorn et al. 2007). These children need to continuously develop and improve their skills, which can be enhanced through meaningful educational practices (Bildiren and Kargın 2019; Pfeiffer 2017). 

In relation to the 26 articles reviewed, it was found that 17 of them specifically referred to educational programs, provisions, or strategies. Some of these were guidelines on what is needed, while others were based on the opinions of teachers and the implementation of particular strategies. Five articles focused on identification processes, characterization of IP learners, and proposals for socioemotional development strategies, and four of the studies mentioned the need to address the educational realm but did not provide guidance on how to do so.

**Early Intervention**

The studies that referred to the fact that interventions with IP children should be carried out early in their development (n = 10) agreed that this type of intervention helps children recognize their abilities, improve the level of their skills, support their development, and prevent developmental risk factors (e.g., Aldosari 2023; Bildiren and Kargın 2019; Darga and Ataman 2021; Kroesbergen et al. 2016; Sofologi et al. 2022). In Bildiren and Kargın’s (2019) research, for example, an early intervention program using a project-based approach was found to significantly improve knowledge transfer and problem-solving skills in precocious learners. The evaluation of the implementation involved pre- and post-assessments using a Problem-Solving Skills Scale, as well as a follow-up assessment to determine the lasting impact of the program.

In a study with teachers, Cosar et al. (2015) suggested that teachers who work with these children believe that educational interventions should begin at preschool age since their intelligence develops faster and character formation begins.

Early educational experiences have positive long-term effects on both academic and socioemotional learning (Mollenkopf et al. 2021). Kettler et al. (2017b) suggest that educational interventions at this level can help children develop skills such as persistence, creative thinking, risk-taking, and higher-level thinking that are relevant to their future learning and achievement. Their study was conducted in 263 preschool centers through a Preschool Gifted Education Survey to gather information on gifted education services. It is, therefore, relevant to suggest that IP learners need special and challenging activities, as the traditional curriculum is not enough to prevent boredom and stimulate cognitive growth (e.g., Aldosari 2023; Mollenkopf et al. 2021). According to Kroesbergen et al. (2016), teachers should give gifted children more challenging work that matches their high abilities to keep them interested and help them learn more. However, this requires educational provisions for students at this level, which are recognized as critical but scarce (Aldosari 2023; Dereli and Deli 2022; Firat and Bildiren 2023). In their study on the effects of a problem-solving program in gifted preschools, Bildiren and Kargın (2019) found that without the appropriate educational interventions, gifted children may not reach their full potential. This situation needs to be taken seriously considering the findings of Kaplan and Hertzog (2016), which reinforce the idea that the early years of development are crucial for later cognitive, emotional, and social development.

**Learning Environment**

Eight of the reviewed articles referred to this subcategory. Papadopoulos (2020a, 2021) states that professionals working with young gifted children need to create an environment that supports the strengths of IP learners, which includes acceptance and recognition. This author points out that research has shown that gifted children who receive educational services alongside other gifted children experience less loneliness, have more friends, and possess a more positive self-image. Villuendas-Rey et al. (2020) emphasized the crucial role of the learning environment in understanding the child’s relationship with their educational environment, their level of interest and participation in group play, and their tendency to interact with other children.

The learning environment for young gifted children should be enriched and have a variety of activities and materials (e.g., Dereli and Deli 2022; Kettler et al. 2017a; Mohamed and Elhoweris 2022). Specifically, Dereli and Deli (2022) point out the importance of variety in learning centers, the arrangement of the physical environment, group work/peer interaction, and materials such as mind and intelligence games, as well as a well-equipped laboratory. This can have a positive impact on the development of skills such as problem-solving skills (Bildiren and Kargın 2019). In preschool, gifted children need to know that adults support and value their activities, which also happens with their peers, because although they express a preference for working alone, their interest and willingness to work in groups increases when they feel that they can be supported and recognized by their peers (Podobnik and Selan 2021).

**Educational Interventions**

(a) Modification of the curriculum: mentioned in seven articles. Some authors suggest modifying the curriculum to address the advanced learning needs of this group. Modifications can be made by complexifying and expanding the content, as well as providing challenging and advanced learning materials that match young gifted children’s abilities and interests (Daǧlioǧlu and Suveren 2013; Artiles 2022). Differentiation is also mentioned through methodological strategies such as cooperative or project-based learning, workshops, camps, mentoring, online programs (Artiles 2022), and problem-solving skills (Bildiren and Kargın 2019). Also, asking more complex questions (Antoun et al. 2022). Students with strong interests in specific subjects, such as science or art, should have opportunities to explore these areas in depth (Aldosari 2023).
(b) Enrichment: Mentioned in seven of the reviewed articles, it allows for the inclusion of additional content and greater depth in the curriculum in areas of interest (e.g., Dereli and Deli 2022). Darga and Ataman (2021) evaluated the effect of applying an enrichment program to first grade gifted students and their typically developing peers by means of a pre-test and post-program post-test and found that scores increased in the post-test measurement in both groups. It is suggested that enrichment for gifted students and their peers is a strategy that improves the performance and development of all children.
(c) Acceleration: Mentioned in five articles, it allows gifted children to move through the curriculum faster so that they can progress more quickly in school (e.g., Aldosari 2023). It is recognized as a type of individual response, and examples given are grade skipping (e.g., Artiles 2022) and curriculum compacting (e.g., Antoun et al. 2022; Artiles 2022; Mohamed and Elhoweris 2022).

Another relevant aspect identified in this review relates to the barriers to educational implementation due to the lack of clear policies or resources, in addition to the need for teacher training (Darga and Ataman 2021). The lack of teacher training leads to erroneous and conflicting beliefs, which can create complex challenges when implementing educational interventions (Jawabreh et al. 2022; Mohamed and Elhoweris 2022). This lack of training is linked to IP being one of the most neglected fields of study in gifted education, as noted by Hertzog et al. (2018), Jolly and Kettler (2008), Martins and Chacon (2016), and Walsh (2014). Teacher training is essential for effectively supporting gifted students (Aldosari 2023).

## 4. Strengths and Limitations of This Review

One of the main strengths of the present study is its novelty, as no systematic reviews focusing on IP have been found to date, particularly in the last decade. In addition, this review included countries that have not always been represented in research on precociousness and giftedness, such as Cuba and Palestine.

It is important to note that although this is not an exhaustive review, it provides a first approximation of what has been achieved empirically in the field of IP. This review offers an overview of the characteristics of this group that are consistent across different international samples, as well as interventions for precocious learners that consider the most optimal ways of working at the preschool level.

In terms of limitations, this is a limited review, as although precocity has been a much-discussed topic over the past decades (i.e., longitudinal studies), effective interventions for this group have not necessarily been investigated. 

The decision to limit the search to peer reviewed publications in English and Spanish is another limitation that may have introduced bias by leaving out publications in other languages.

It is important to note that this review does not include other works included in the gray literature (e.g., dissertations, conference proceedings, and book chapters), which has also been found to make positive contributions to theory and practice (Adams et al. 2016).

Finally, the selection of certain search terms over others can also be considered within the limitations of this systematic review. For example, what refers to IP characteristics (versus traits for example), as well as limiting the search only to the concept of “programs” (versus other terms such as interventions). The underlying decisions aimed to perform a narrow and focused search, but it is certainly recognized that adopting certain terms over others may introduce biases into the results.

## 5. Conclusions and Directions for Future Studies

One of the research objectives of this review was to address the conceptualizations of precocity that underpin each of the included studies. Although an approximation and/or definition of the concept can be found in most of the studies, the authors provide a general overview of the construct of giftedness, its main characteristics, and historical discussions around the concept in general. However, they often do not make specific references from these definitions to precocity, despite precocity being a robust field, especially in the last 50 years (Lubinski and Benbow 2021).

Although the field of giftedness is still underpinned by theoretical discussions, it is important that empirical research adopts conceptualizations that are culturally relevant and appropriate to the context in which they are developed, as mentioned by Carrillo (2013), Ngara (2008), and Plucker et al. (2021). This can also help to ensure that findings can be interpreted in light of these definitions. It is important to have holistic definitions that integrate multiple components of precocity and giftedness, including often-overlooked intersectional elements such as race and gender. Considering diversity is crucial in this field, as it has significant implications for the identification process of young gifted learners. Therefore, such an approach needs to move away from the traditional perspective (e.g., IQ) towards an integrative definition that includes the measurement of other skills (e.g., motor, non-verbal, artistic), direct classroom observation, and incorporating the voices, experiences, and insights of the IP learners themselves.

Another issue arising from this systematic review regarding the conceptualization of IP is the lack of comprehensive models that understand it as more than just an assemblage of characteristics. It should be viewed as a progression that may or may not culminate in giftedness but nevertheless requires systematic attention over time. However, if there is a lack of knowledge about IP and its manifestations, it will be difficult to consider models (i.e., Gagné 2015) that allow for understanding it as a construct that develops over time, for which it needs individuals and entities that can play a critical role in this process. 

Regarding the second research objective, the characteristics that can be distinguished in this group of boys and girls, the studies reviewed emphasize cognitive traits, which is supported by the use of instruments that measure them. These include greater speed and accuracy in reasoning, deep perception, which implies sharper and more detailed observation, and a larger vocabulary. This last aspect is also mentioned by authors such as Lubinski and Benbow (2021) when they indicate that verbal skills are one of the essential traits of IP learners. In addition, executive functioning is revealed, highlighting working memory, cognitive flexibility, inhibitory control, and planning in relation to age and gender peers. This aligns with the study by Sastre-Riba and Castelló-Tarrida (2017), which found that executive functions are more advanced in intellectually gifted children compared to their peers of the same age.

On the other hand, it is noted that there is less consensus in the literature regarding artistic, creative, motor, and socioemotional skills as early indicators of high ability. The above suggests the importance of generating studies, both nationally and internationally, that take these skills into account to determine their relevance in the early development of IP learners. Also, it would be interesting to address the differences in children’s characteristics according to sex, identifying which skills are more predominant in girls and/or boys, which could be a relevant factor to orient an educational policy that considers the gender perspective at early stages of development. 

Finally, while several cross-sectional studies contribute valuable insights into the characteristics and interventions for early childhood, it is also important to conduct longitudinal research in this area. Such research should include the educational trajectories of IP learners and their contexts. This approach would allow for focus not only on the individual but also on other systems, such as the family and the school, highlighting the relevance of these contexts in creating opportunities for skill development. 

Regarding the third research objective about the services provided for IP learners, the reviewed articles consistently emphasize the need for creating environments and conditions that foster skill development, underscoring the importance of specialized educational programs for precocious children (e.g., Artiles 2022). Adequate attention would allow for avoiding disenchantment, boredom, and socioemotional difficulties, as indicated by Kroesbergen et al. (2016) and Mathijssen et al. (2021).

Gómez and Mir (2011) indicate that there are three types of measures that can be included in the educational responses for this group: (a) ordinary, (b) extraordinary, and (c) exceptional. The first ones correspond to adaptations that can be made by the teacher in the classroom. The second ones refer to curricular expansion, enrichment of curricular content, as well as the development of creativity, metacognition, motivation, interest, effort, and social skills, among others. The third measure refers to acceleration and grouping. Both extraordinary and exceptional measures can be formalized in individualized plans that describe the set of supports and adaptations that children will require. Most of the reviewed articles on IP can be situated on type (b), extraordinary educational responses, such as enrichment, curricular differentiation, methodological modification, and acceleration (García-Martínez et al. 2021). Regarding exceptional responses, particularly acceleration, the reviewed articles typically refer to advancing courses and compacting the curriculum. However, they often overlook early entrance, which is specifically relevant to this group. Early entrance has established guidelines for implementation (see Lupkowski-Shoplik et al. 2018) and represents an important resource for precocious children who may face an educational path that fails to meet their needs when dealing with an undemanding educational system. Although there is a greater tendency to present curriculum development strategies, according to Papadopoulos (2020b), the stimulation of the social–emotional competencies of IP children should be considered, since they improve their self-perception of school competence, self-perception of physical competence, and self-perception of relationships with peers.

Lastly, it is important to mention that only one of the studies meeting the inclusion criteria for analysis was conducted in a Spanish-speaking country. Additionally, most of the research was conducted in European and Asian countries. Thus, it can be argued that research on the topic of IP has advanced, and this knowledge can be applied to other areas. 

Following the same idea, stable collaboration networks become relevant to investigate and propose improvements to the identification and educational attention required by IP learners. This is supported by the results of the study by Hernández-Torrano and Kuzhabekova (2020), from which it is concluded that there are few working links between countries, organizations, universities, and researchers to analyze and promote the subject of giftedness in general and IP in particular, contemplating spaces for inquiry contextualized to the educational, cultural, and social contexts of IP children.

## 6. Implications for Educational Practice

In terms of the practical implications of this research, it is important to note the critical need for adequate funding, policies, and support for this group of children. In particular, the following guidelines are proposed that can inform future practices in IP, based on the results of this systematic review:

**Early Identification and Assessment**

Identifying IP learners at an early age is essential, as highlighted by Villuendas-Rey et al. (2020). Conducting holistic assessments of children’s skills is critical for accurate identification. However, one must carefully select identification procedures for IP children, considering the social, educational, and cultural context of the children.

**Educational Programs and Involvement**

Studies suggest the importance of having an adequate curriculum, learning environment, and knowledge on the part of educators and parents. To achieve this, specialized educational programs must involve parents and specialized teachers to offer challenging activities that allow children to apply and develop their skills. At the classroom level, educators can consider several approaches, including the following:

Acceleration: Allowing gifted learners to move more quickly through the curriculum.
Enrichment Programs: Providing comprehensive immersion in specific areas of interest.
Both approaches require teachers specifically trained to educate intellectually precocious children.

**Research and Development**

The findings clearly indicate the need for more research on IP and educational strategies, particularly focusing on how precocity is conceptualized and understood, its characteristics, and ways to enhance teaching and learning processes. While the current literature predominantly addresses identification processes, there is a need for studies exploring specific enrichment experiences and their positive impacts on IP learners.

**Specialized Educational Initiatives**

It is very important to improve educational and developmental opportunities for IP children in preschools and, likewise, build a solid educational foundation for children who are transitioning to primary school. This highlights the importance of creating specialized educational initiatives, interventions, and support systems aimed at catering to the distinctive requirements of intellectually gifted children. By addressing these needs, educational practices can better support the development and success of young gifted learners.

## Figures and Tables

**Table 1:** Inclusion and exclusion criteria.

Categories	Criteria	
	Inclusion	Exclusion
Participants	Preschool teachers of young gifted children	Considered only secondary education teachers
	Young gifted children that were less than or equal to 6 years old in the sample	Considered only gifted children over 6 years old
	Parents of young gifted children	
Concept	Refers to the concept of giftedness in young gifted children	Reports on educational policies
	Reports on the characteristics of giftedness in young gifted children	
	Reports on giftedness provision in young gifted children	
Context	Is carried out in a preschool educational context	It is carried out in a non-educational context
	Is carried out in a non-formal preschool educational context	

**Table 2:** Categories and subcategories of analysis.

Category	Subcategory	Total Number of Studies
Conceptualizations of giftedness and precocity	Giftedness as abilities or traits	11
	Broad definition giftedness	7
	Giftedness as a multifactorial/developmental construct	3
Characteristics of intellectual precocity	Cognitive characteristics	3
	Motor skills	23
	Linguistic development	12
	Artistic/creativity skills	9
	Socioemotional skills	26
Programs and modalities of educational interventions	Early intervention	10
	Learning environment	8
	Educational interventions	19

**Table 3:** List of articles selected for review.

N°	Title	Authors	Year of Publication	Journal	Location	Method Synthesis	Participants					
							Children	Age	Parents	Age	Teachers	Years of Experience as Preschool Teachers
1	Gifted Children through the Eyes of Their Parents: Talents, Social-Emotional Challenges, and Educational Strategies from Preschool through Middle School.	Renati R.; Bonfiglio N.S.; Dilda M.; Mascia M.L.; Penna M.P.	2023	*Children*	Italy	Mixed-method	44 gifted children	4 children were 5 years old, 16 children 6–7 years old, 16 children 8–10 years old, and 8 children 11–14 years old	44 families of gifted children	43 mothers with a mean age of 42 years old. 44 fathers with a mean age of 45.2 years old		
2	The characteristics of gifted children with learning disabilities according to preschool teachers.	Firat T.; Bildiren A.	2023	*Early Years: An International Journal of Research and Development*	Turkey	Qualitative: case study					41 preschool teachers working in different schools in a southern province in Eastern Turkey participated in the study	Seven of the teachers had 0–5 years of teaching experience, ten had 5–10 years, nine had 10–15 years, eight had 15–20 years, and seven had over 20
3	Exploring the Characteristics of Gifted Pre-School Children: Teachers’ Perceptions.	Jawabreh R.; Danju İ.; Salha S.	2022	*Sustainability*	Palestine	Mixed-methods					450 female preschool teachers with less than 3 years and up to a little more than 6 years of experience working with female preschool	
4	The Gifted Rating Scales-Preschool/Kindergarten Form (GRS-P): A Preliminary Examination of Their Psychometric Properties in Two Greek Samples.	Sofologi M.; Papantoniou G.; Avgita T.; Lyraki A.; Thomaidou C.; Zaragas H.; Ntritsos G.; Varsamis P.; Staikopoulos K.; Kougioumtzis G.; Papantoniou A.; Moraitou D.	2022	*Diagnostics*	Greece	Quantitative	Study 1 107 kindergarten students (55 girls and 52 boys) Study 2 26 kindergarten children (12 boys and 14 girls)	Mean age in years: 5, 5 Mean age in years: 5, 7			Study 1 107 kindergarten teachers (105 female and 2 men; mean age = 43.01 years) Study 2 3 kindergarten teachers	
5	Pre-school teachers’ knowledge and needs related to noticing gifted children and the enrichment model.	Dereli E.; Deli H.	2022	*Dergi Park Akademik*	Different regions of Turkey	Qualitative: case study and interviews					30 preschool teachers	14 of the participants had 1–4 years of teaching experience; 6 had 5–9 years; 7 preschool teachers had more than 10 years of experience
6	Children with high intellectual abilities.	Artiles, C. A.	2022	*Pediatria Integral*	Kazakhsta	Bibliometric approach	WOS and Scopus databases	Does not apply				
7	Gifted Education in Lebanon: Time to Rethink Teaching the Gifted.	Antoun M.; Plunkett M.; Kronborg L.	2022	*Roeper Review*	Lebanon	Mixed study with a case study design					280 teachers	
8	Perceptions of preschool teachers of the characteristics of gifted learners in Abu Dhabi: A qualitative study.	Mohamed A.; Elhoweris H.	2022	*Frontiers in Psychology*	Abu Dhabi, United Arab Emirates	Qualitative interviews					16 preschool teachers (13 teachers were homeroom teachers and 3 were special education teachers working in preschool)	The participants had 3 to 14 years of experience as preschool teachers
9	Relationship between Pre-Schoolers’ self-regulation, language, and early academic skills: The mediating role of self-regulation and moderating role of gender	Gözüm, A.İ.C., Aktulun, Ö.U	2021	*Current Psychology*	Afyonkarahisar and Kars, Turkey	Quantitative	363 children				20 preschool teachers (15 teachers were female, and 5 teachers were male)	5 participants had between 3 and 6 years of experience as preschool teachers; 9 teachers had 7–10 years; and 6 teachers had 10–15 years
10	Exploring public and private preschool teachers’ beliefs and practices regarding gifted children from three to six years old in Riyadh, Saudi Arabia.	Aldosari D.H.	2021	*Early Years*	Riad and Riyadh Saudi Arabia	Qualitative method: interview					7 Preschool teachers (3 public and 4 private preschool teachers)	
11	Searching for a more valid form of parental rating scales of preschoolers’ intellectual giftedness—development and validation of the preschooler’s ability rating scale (Pars).	Jabůrek M.; Cígler H.; Portešová Š.; Ťápal A	2021	*Ceskoslovenska Psychologie*	Czechoslovakia	Quantitative	147 children	6 (23%) of the sampled children were 4 year old, 110 (42%) were 5 year olds, and 93 (35%) were 6 year olds		277 parents (263 mothers and 14 fathers)		
12	Emerging School Readiness Profiles: Motor Skills Matter for Cognitive- and Non-cognitive First Grade School Outcomes.	Kamphorst E.; Cantell M.; Van Der Veer G.; Minnaert A.; Houwen S.	2021	*Frontiers in Psychology*	Participants resided in the northern part of the Netherlands	Quantitative	47 Preschool education children	Mean age of 3, 4 years old	Parents of preschool education children	There is no information		
13	The effect of class-wide enrichment applied to gifted and normal children in early childhood.	Darga H.; Ataman A.	2021	*Participatory Educational Research* (PER)	Turkey	Screening model and experimental design	477 students of which 35 identified as gifted	6 years old				
14	Testing, Identifying, and Serving Gifted Children With and Without Disabilities: A Multi-State Parental Perspective.	Mollenkopf D.L.; Matyo-Cepero J.; Lewis J.D.; Irwin B.A.; Joy J.	2021	*Gifted Child Today*	The respondents represented 38 of the 50 states of the United States	Quantitative			177 Parents of gifted children or twice-exceptional children	Not specified		
15	Visual Art Gifted Child in Pre-School and Early School Years.	Podobnik U.; Selan J.	2021	*Creativity. Theories–Research–Applications*	Slovenia	Qualitative	Girl with artistic talent	3, 6 years old				
16	Examining the relationships among cognitive ability, domain-specific self-concept, and behavioral self-esteem of gifted children aged 5–6 years: A cross-sectional study	Papadopoulos D.	2021	*Journal of behavioral Sciences*	Athens, Greece	Quantitative	108 Gifted children (59 boys and 49 girls)	5–6 years old				
17	Psychological framework for gifted children’s cognitive and socioemotional development: A review of the research literature and implications.	Papadopoulos D.	2020	*Journal for the Education of Gifted Young Scientists*	Greece	A review of the research literature	Does not apply					
18	Prediction of high capabilities in the development of kindergarten children.	Villuendas-Rey Y.; Rey-Benguría C.F.; Camacho-Nieto O.; Yáñez-Márquez C.	2020	*Applied Sciences*	Cuba	Mixed-method	1032 children, of them, 91 were marked as having high potential	5 years old				
19	The Saudi version of the gifted and talented checklist for parents: An instrument for rating the characteristics of gifted kindergarten children.	Almerab M.M.; Bakhiet S.F.A.	2020	*High Ability Studies*	Riyadh, Saudi Arabia	Quantitative			615 parents of preschool children participated in the study: 599 mothers and 16 fathers			
20	The state and development of research in the field of gifted education over 60 years: A bibliometric study of four gifted education journals (1957–2017).	Hernández-Torrano D.; Kuzhabekova A.	2020	*High Ability Studies*	Nur-Sultan, Kazakhstan	Descriptive bibliometric study	Two bibliographic databases: Web of Science and Scopus	Does not apply				
21	Effects of a social-emotional learning-based program on self-esteem and self-perception of gifted kindergarten students: A pilot study.	Papadopoulos D.	2020	*Dergi Park Akademik*	Greece	Quantitative experimental design	120 Gifted kindergarten students	Aged 5 to 6 years				
22	The effects of project-based approach in early intervention program on the problem solving ability of gifted children.	Bildiren A.; Kargın T.	2019	*TED EĞİTİM VE BİLİM*	Izmir, Turkey	Quantitative	114 Potential and gifted children in the preschool period. The final sample was made up of 23 children from the experimental group and 21 children from the control group	Aged 3 to 6 years				
23	Gifted Education in Preschool: Perceived Barriers and Benefits of Program Development.	Kettler T.; Oveross M.E.; Bishop J.C.	2017	*Journal of Research in Childhood Education*	Southern state in the United States	Qualitative			Directors and assistant directors of 263 private preschools: 150 general child care centers; 97 preschool education programs; 19 accredited Montessori preschools; 13 in-home preschools; 29 others	Not specified		
24	The Psychological Well-Being of Early Identified Gifted Children.	Kroesbergen E.H.; van Hooijdonk M.; Van Viersen S.; Middel-Lallema M.M.N.; Reijnders J.J.W.	2016	*Gifted Child Quarterly*	Holland	Cross-sectional descriptive field study	35 gifted primary school children 34 typically developing children	Aged 6 to 8 years				
25	Investigating the preschool training for gifted and talented students on gifted school teachers’ view.	Cosar G.; Cetinkaya C.; Cetinkaya C.	2015	*Journal for the Education of Gifted Young Scientists*	Balikesi, Turkey	Qualitative: case study					10 gifted teachers (8 female and 2 male)	Teachers had an average of 10.5 years of experience working with preschool children
26	The role of teacher and family opinions in identifying gifted kindergarten children and the consistence of these views with children’s actual performance.	Daǧlioǧlu H.E.; Suveren S.	2013	*Educational Sciences: Theory and Practice*	Turkey	Quantitative	113 gifted preschool children	5–6 years	Parents of gifted children		Preschool teachers	

## Data Availability

No new data were created or analyzed in this study. Data sharing does not apply to this article.
